# Social buffering of oxidative stress and cortisol in an endemic cyprinid fish

**DOI:** 10.1038/s41598-023-47926-8

**Published:** 2023-11-23

**Authors:** Sophia Schumann, Gloria Mozzi, Elisabetta Piva, Alessandro Devigili, Elena Negrato, Andrea Marion, Daniela Bertotto, Gianfranco Santovito

**Affiliations:** 1 Department of Biology, University of Padova, Via Ugo Bassi 58E, 35131 Padova, Italy; 2 Department of Environment, Land and Infrastructure Engineering, Politecnico di Torino, 10129 Torino, Italy; 3 Department of Comparative Biomedicine and Food Science, University of Padova, 35020 Padua, Italy; 4 Department of Industrial Engineering, University of Padova, 35131 Padua, Italy

**Keywords:** Conservation biology, Ecophysiology, Biomarkers, Animal behaviour, Animal physiology, Ichthyology

## Abstract

Fish exhibit complex social behaviours that can influence their stress levels and well-being. However, little is known about the link between social interactions and stress in wild fish, especially in running water environments. While many studies have explored the stress axis in fish, most have focused on specific social contexts, leaving gaps in understanding stress responses to social changes. Our study investigated collective behaviour and stress in wild Italian riffle dace (*Telestes muticellus*) in a controlled experimental setup simulating a natural river system. Results reveal that group-living fish have lower cortisol and oxidative stress levels in muscle tissue compared to solitary counterparts, suggesting a calming effect of conspecific presence. Additionally, we observed upregulated expression of antioxidant enzymes in group-living fish, indicating potential benefits to antioxidant defence systems. These insights shed light on the dynamic relationship between group behaviour and stress in wild fish within running water habitats and emphasise the use of multidisciplinary approaches.

## Introduction

Sociality, or the tendency of animals to form groups, is a ubiquitous phenomenon observed across the animal kingdom, from small insects to large mammals, including fishes. The complex interplay among ecological, genetic, and behavioural factors has shaped the evolution of animal sociality^1^. It has developed a remarkable diversity of social structures, from loosely organised aggregations to highly structured societies with complex communication and division of labour^1, 2^. Collective behaviour in fish is a complex phenomenon, shaped by both environmental cues and social interactions, that gives rise to emergent properties of the group^3^. Fish exhibit various social and ecological dynamics, such as schooling and shoaling, which are essential for survival and reproduction^4–6^. The collective dynamics observed in some fish species are characterised by sudden, highly coordinated activities that emerge from a shared experience among group members, fostering a sense of common interest and identity^7^. Unlike the continuous schooling behaviour seen in many species, these episodic events are marked by distinct beginnings and endings.

Any change in group dynamics passes through a modification of individual swimming, a crucial aspect of fish behaviour and physiology, which is, in turn, affected by social interactions^8^. The swimming ability of fish can be modulated by the stress response, a complex physiological and behavioural reaction triggered by a range of factors, including social changes such as alterations in social status and group composition and environmental conditions^9–11^. The stress response is a crucial adaptive mechanism for survival, but excessive or prolonged activation of this response can negatively impact health and welfare^12^. Thus, antagonistic social behaviour in fish can be a significant source of stress, affecting the welfare of individuals and the functioning of groups^11^.

In general, disruptions to social interactions and group dynamics, for example, due to perceived predation risk or pollution^13, 14^, can trigger the activation of the stress response in fish, especially when they attempt to establish new social roles and relationships within the group^9^. Social activities can be affected by many factors, such as natural environmental changes in water temperature and quality, or external anthropogenic impacts, such as acoustic stress, which lead to changes in social behaviour within the group^15–17^. Moreover, the limited availability of resources such as shelter and food can often result in increased competition and aggression due to disruption of the social hierarchy, which affects stress^9, 18, 19^. The stability of social structures is a critical factor in shaping collective behaviour in fish and understanding its influence is essential for maintaining optimal welfare in captive and wild populations^6^.

While social structures and hierarchies in fish have been extensively studied in the context of stress, less emphasis has been placed on exploring the beneficial effects of conspecific availability in reducing stress within group living dynamics^20^. Group living can provide benefits such as increased vigilance, better predator detection, and improved hydrodynamics, all aspects which may indirectly affect individual stress^21^. By understanding the factors contributing to stress reduction in fish, we can develop effective management strategies that prioritise the formation of stable social groups^20, 22^. This can enhance social buffering, the phenomenon by which stress level is reduced by group living^23^ and which is particularly important in uncertain or dangerous environments.

While previous studies have explored the influences of social interaction and stress in fish in artificial settings^9, 11, 15, 22, 24–26^, limited attention has been given to investigating the impact in their natural settings as characterised by running water^27^.

Riverine environments exhibit a broad spectrum of flow velocities, and our understanding of group dynamics within such environments remains relatively limited. This knowledge gap is particularly significant because flow velocity substantially influences fish behaviour and physiology. For instance, hydrodynamics has been demonstrated to play a pivotal role in shaping the structural organisation of fish schools^28^, thereby influencing information exchange among individuals^29^. Additionally, flow velocity can have a pronounced impact on fish stress^27, 30^. However, the intricate relationship between collective behaviour and fish stress in diverse flow conditions remains unexplored. Understanding these effects holds promise for optimising environmental enrichment strategies, which is essential for managing fish health and behaviour in both natural and artificial settings^29^. Potentially, multi-biomarker approaches, including oxidative stress damage and antioxidant enzyme gene expression, offer a comprehensive understanding of the underlying mechanisms that sustain the adaptive consequences of collective behaviour^30^.

Commonly, cortisol is used widely as a biomarker to assess the stress response in fish. Recent studies have suggested that collective behaviour in fish, such as group living or schooling, can significantly influence body cortisol levels and help reduce the negative impacts of stress on individuals^31^. Nevertheless, the rapid change of cortisol release in the blood makes it sometimes challenging to exclude the potential impacts of other factors, such as the handling of specimens during sampling, on the fish stress response^32^. Alternative approaches could be analysing different matrices^33^, for example, measuring cortisol in the muscle, which might increase with a time shift due to muscle activity. The presence of other steroids, including sex hormones, in various matrices such as mucus, gut, and muscle has been reported, indicating the ability of cortisol to diffuse through cell membranes and distribute to multiple tissues^34–37^.

Additionally, using other physiological markers such as oxidative stress^10, 11, 25, 38^ provides a more reliable measurement and shows both early and cumulative effects of stress over time. Oxidative stress refers to an imbalance in the production of reactive oxygen species (ROS) and the action of the antioxidant defence system, leading to disruption and harm in the function of biomolecules such as proteins, lipids, and DNA^39^. Antioxidant enzymes are some of the most essential components of the cellular defence system against oxidative stress. In fish, these include superoxide dismutase (SOD), catalase (CAT), glutathione peroxidase (GPx), and peroxiredoxins (Prdx), often characterised by the presence of multiple isoforms whose expression (both number and types) depends on the species, tissue, and environmental conditions such as temperature, salinity, or chemistry^40–42^.

In recent years, several studies pointed out the connection between social hierarchies and oxidative stress capacity in fish^11^, where it was shown that enhanced oxidative stress levels were linked to higher social aggression^25, 38^. Exploring oxidative stress damage in fish often focuses less on behavioural aspects, with limited research delving into the involvement of antioxidant enzymes in these organisms. However, understanding the interplay between oxidative stress, behaviour, and antioxidant defence mechanisms is crucial for comprehending how fish adapt to environmental challenges. In this context, knowledge of the induction kinetics of these enzymes becomes pivotal in understanding the fish’s ability to counteract the adverse effects of oxidative stress^40^. Investigating the relationship between oxidative stress damage and antioxidant enzyme expression in fish is essential for obtaining a holistic understanding of the mechanisms that maintain physiological balance and ensure the proper functioning of fish in the presence of various stressors^43^. Studying the impact of social interactions on these mechanisms can provide insight into the adaptive consequences of collective behaviour in fish. By exploring the relationship among collective behaviour, stress hormones, oxidative stress damage, and anti-stress physiological responses, we aimed to provide valuable insights into the potential impacts of environmental stressors on fish populations and their ability to acclimatise and survive.

In this study, we systematically investigated the effects of group sizes (1, 2, and 6 fishes) on the stress responses in fish after swimming in various flow conditions. By examining behaviour at different velocities and measuring physiological markers afterwards, we gained insights into the complex dynamics of stress adaptation in aquatic ecosystems. Therefore, *Telestes muticellus* which holds ecological importance due to its ubiquity and restricted and fragmented distribution in Italy^44–46^, was tested concerning their natural environment, running water simulating a shallow stream velocity of 35 cm/s^47^. These findings not only enhance our understanding of the interplay between social structure and hydrodynamic conditions but also underscore the pivotal role of water flow velocity in modulating the physiological stress responses of non-typically migrating fish species.

## Results

### The stress level in the muscle tissue

**Figure 1:**
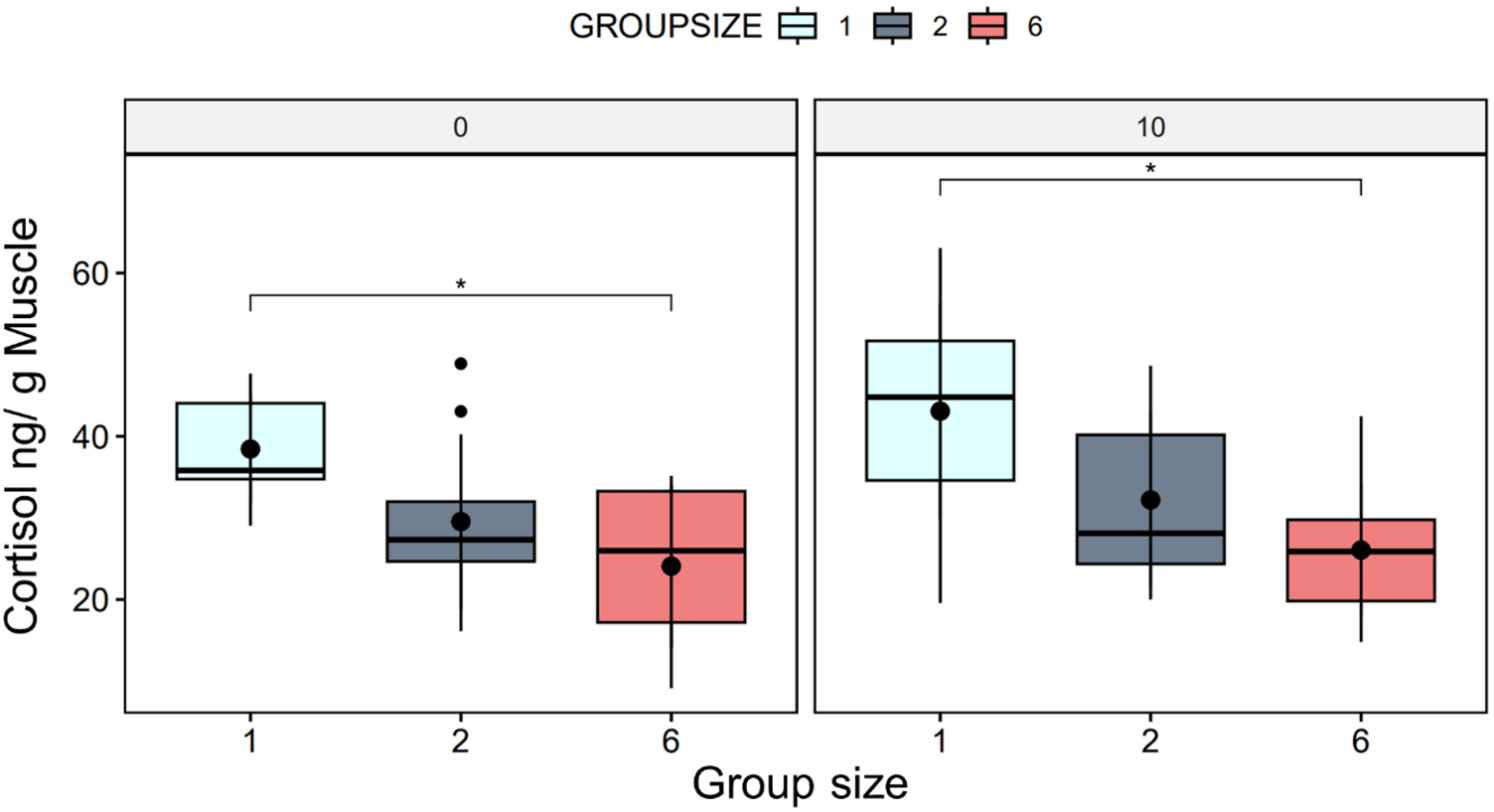
The boxplot shows the cortisol levels measured in fish muscle (ng/g) tissue under different experimental conditions. Numbers over the graphs indicate the time after the end of experiments (0 and 10 min.). The plot indicates significantly low cortisol levels in the two-fish and six-fish groups compared to the single-fish condition (*p* < 0.05) (N = 10 per condition and time point).

Social grouping affected stress levels in fish. Specifically, cortisol levels were found to be significantly lower in fish tested in groups of six compared to solitary fish (*p* < 0.01), with this effect observed both immediately after the experiment and after 10 min (Fig. 1).

**Figure 2:**
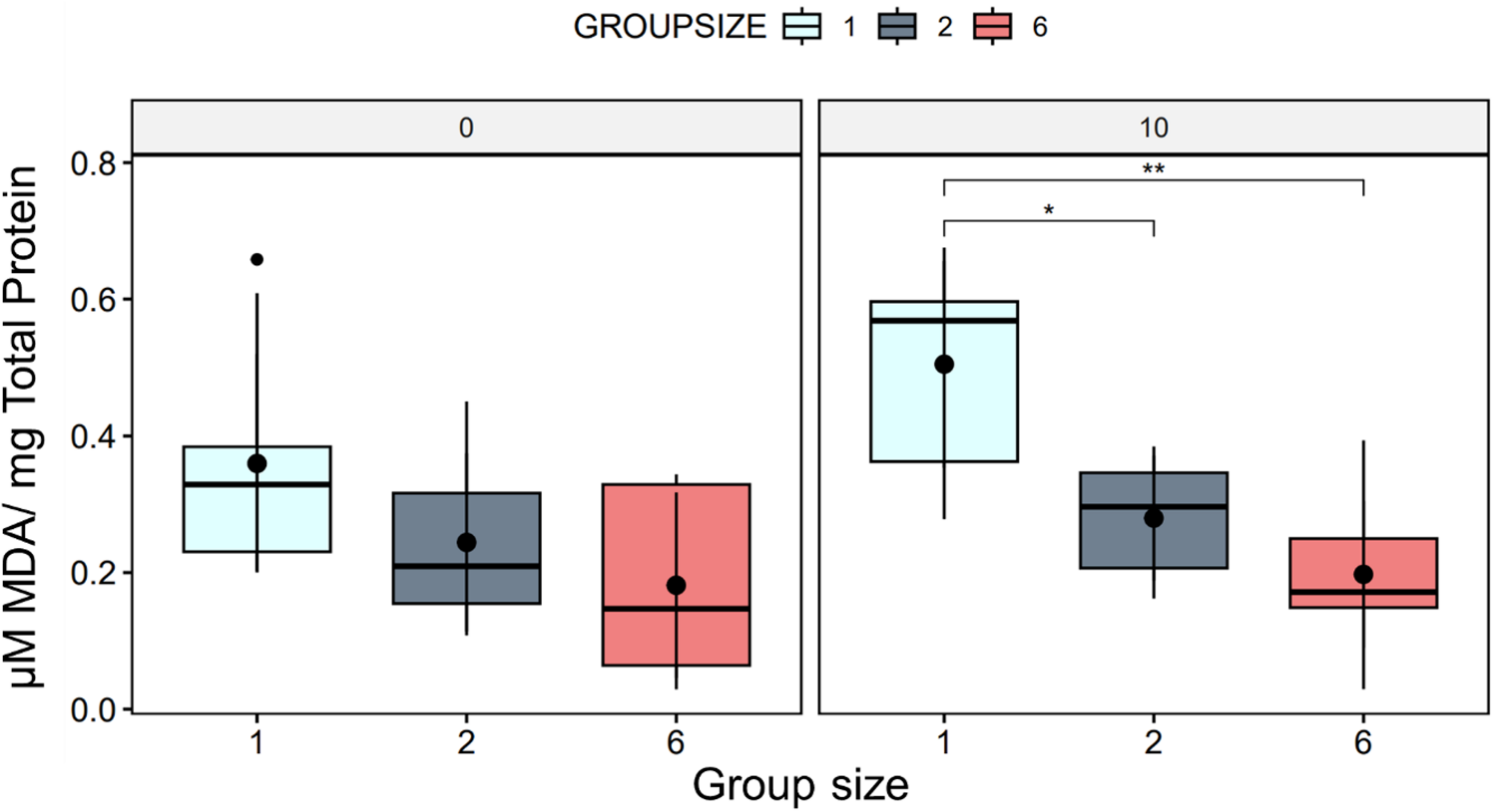
The boxplot displays the distribution of MDA levels measured in fish muscle tissue under different experimental conditions. Numbers over the graphs indicate the time after the end of experiments (0 and 10 min.). The plot indicates no significant difference in MDA levels among the groups without a waiting period. Still, there was a significant increase in MDA levels in single fish compared to the two- and six-fish groups after a waiting period (N = 10 per condition and time point).

No significant difference in malondialdehyde (MDA) levels was observed between solitary fish and the two- and six-fish groups after the initial 30 min of experimental conditions. However, 10 min after the end of the treatment, MDA levels were found to be significantly lower in the two-fish group (*p* > 0.05) and highly significantly lower in the six-fish group compared to fish swimming alone (Fig. 2).

**Figure 3:**
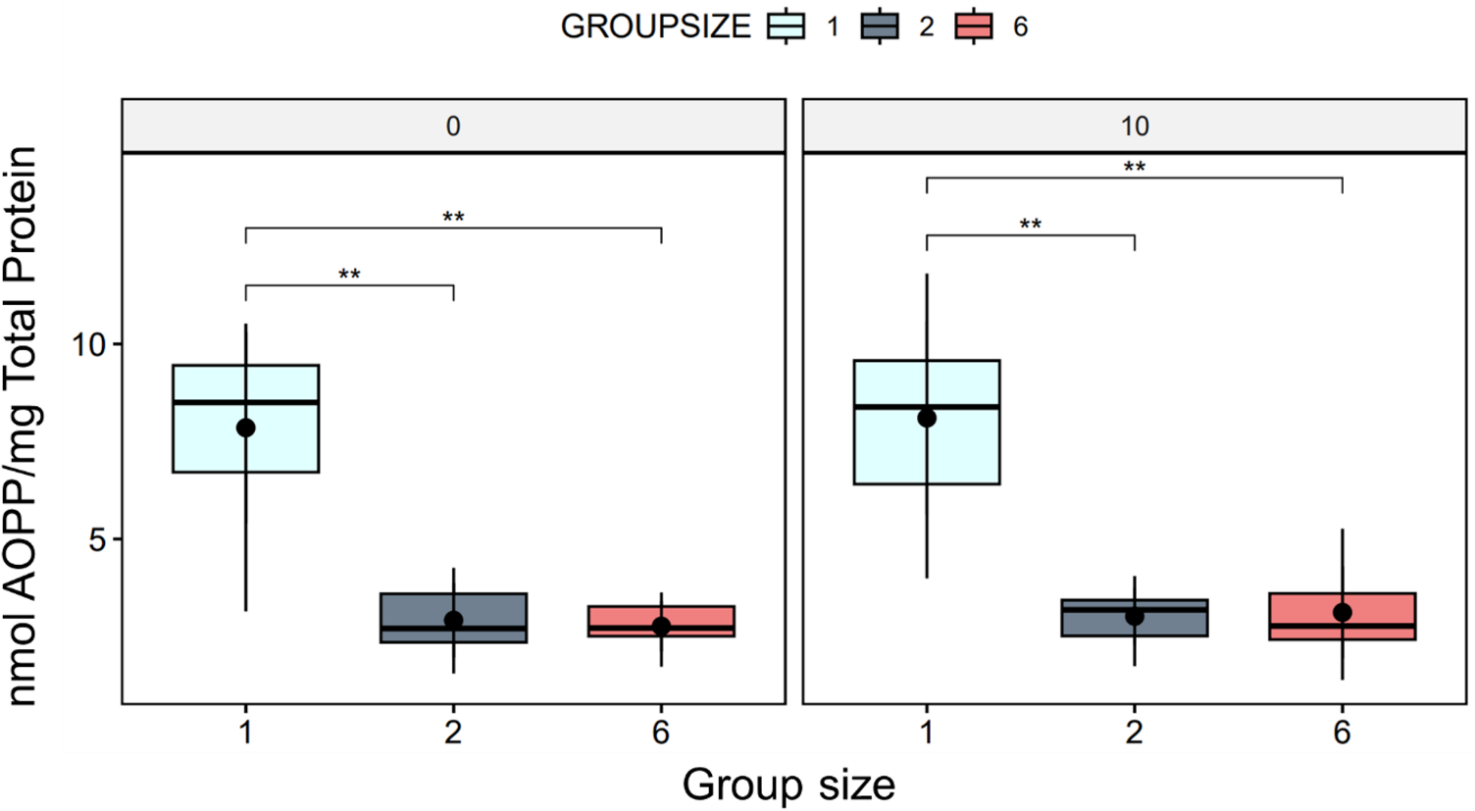
The boxplot shows the distribution of AOPP levels measured in fish muscle tissue under different experimental conditions. Numbers over the graphs indicate the time after the end of experiments (0 and 10 min.). The plot indicates significantly low AOPP levels in the two-fish and six-fish groups compared to the single-fish condition, regardless of whether a waiting period was used (*p* > 0.005) (N = 10 per condition and time point).

Finally, advanced oxidation protein products (AOPP) levels were found to be highly significantly lower (*p* > 0.01) in both the two-fish and six-fish groups, with this effect observed both in the presence and absence of a waiting period relative to the single-fish condition (Fig. 3).

### Expression of antioxidant enzymes

**Figure 4:**
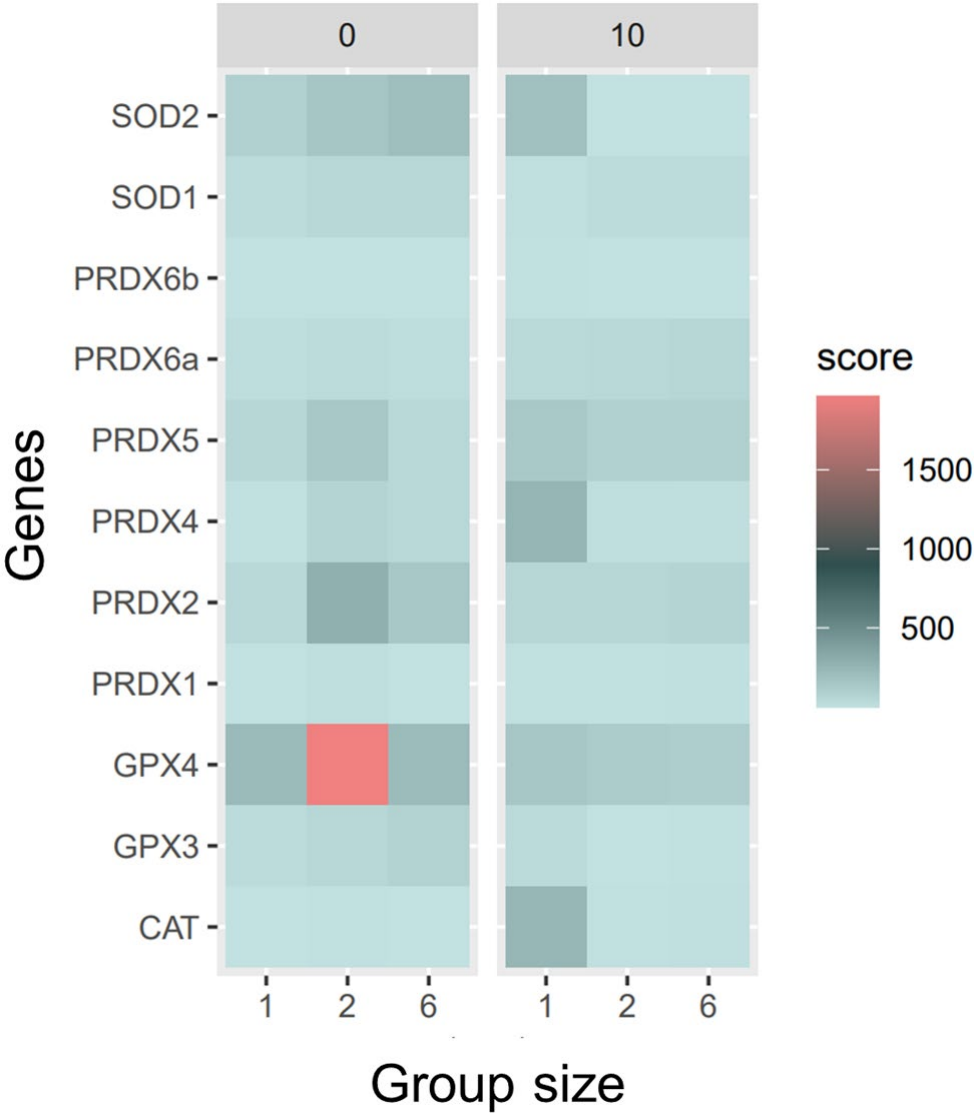
The heatmap was generated to visualise the expression levels of all genes encoding antioxidant enzymes across the different treatments and time points. Each row in the heatmap represents a single gene, while each column represents a specific experimental condition (1, 2, and 6 fish) and time combination. The heatmap colour scale represents the relative expression levels, with red indicating a higher gene expression and green indicating lower expression measured by qPCR. The results show distinct patterns of gene regulation across the different groups and time points. In particular, genes such as *gpx4*, *prdx2*, *prdx4*, and *prdx5* showed higher expression levels in the two-fish treatment than the other treatments. In contrast, after a waiting period, *gpx3, gpx4, prdx4,* and cat showed a significantly higher expression levels in the single-fish treatment (*p* < 0.05). These findings suggest that regulating antioxidant enzyme genes in response to oxidative stress may be influenced by social context and waiting periods in fish. (Additional graph—Figure S3).

The expression of genes encoding antioxidant enzymes was analysed by qPCR to localise oxidative stress balance in the cell (Fig. 4). Our analysis revealed two distinct expression patterns of the selected genes immediately after the end of the experiment and after waiting 10 min. Most genes showed their highest expression immediately after the treatment in both two- and six-fish groups. However, after the waiting time, single fish showed a consistently higher expression than fish groups, with only *sod1* being higher in two- and six-fish conditions.

Notably, in fish directly measured after the treatment, we observed a significant difference in the mRNA expression of *gpx4*, *prdx2*, *prdx4,* and *prdx5* genes, which were transcribed at significantly higher levels in the two-fish group (*p* < 0.01) compared to both single and six-fish treatment. Additionally, *prdx2* and *sod1* mRNAs showed significantly higher levels in two- and six-fish groups than in solitary fish (*p* < 0.05). Only *sod2* was significantly higher in the six-fish group than the two-fish one (*p* < 0.05). After the waiting period, we observed a significant increase (*p* < 0.05) in the mRNA expression of *gpx3*, *gpx4*, and *prdx5* in single fish compared to the two-fish and the six-fish groups, characterised by highly significant increases in *sod2*, *prdx4*, and *cat* activation (*p* < 0.01).

### Video analyses indicate that single fish spend more time on the downstream grid

**Figure 5:**
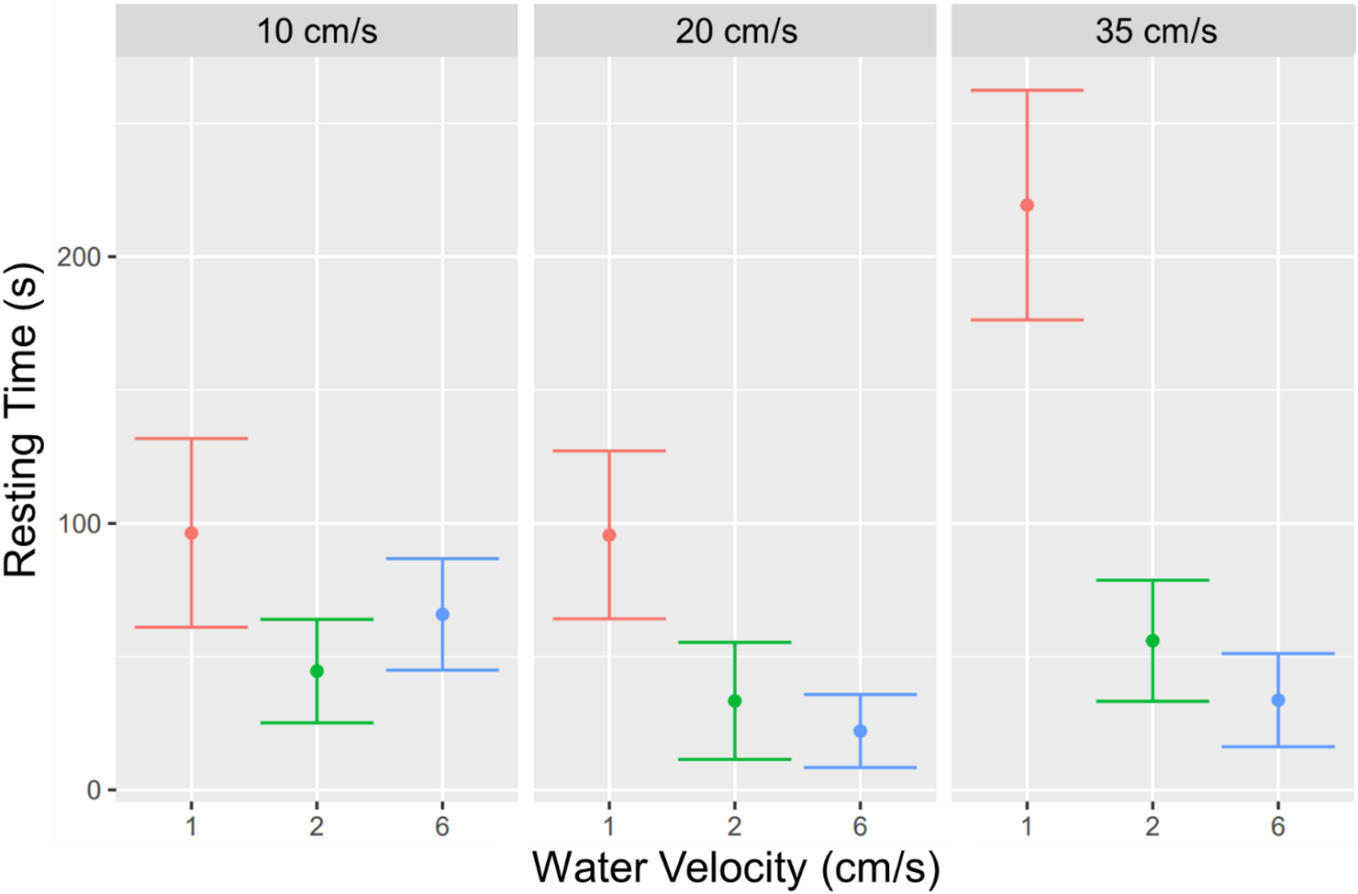
Resting times on the downstream grid: Normalised diagram by the number of total seconds spent on the grid divided by the number of fish. Times were achieved by video observations, showing standard deviation and mean.

In order to analyse swimming motivation, we conducted a detailed analysis of the videos and measured the resting times of fish on the downstream grid of the flume. Our results indicate that the interaction between group size and water velocity influenced time resting on the downstream grid (Fig. 5, *p* < 0.05), with fish resting disproportionally more at fast water velocity when alone. Water velocity per se also seems to have an effect, with fish tendentially resting more at faster water velocities, although the difference is not statistically significant.

In the single-fish test, resting times seem to increase with increasing water velocity, with the fish frequently being pressed to the downstream grid at a speed of 35 cm/s. When the water speed increased, the two-fish and six-fish groups performed significantly better than the one-fish group.

### Exploration of resting times during experiments

**Figure 6:**
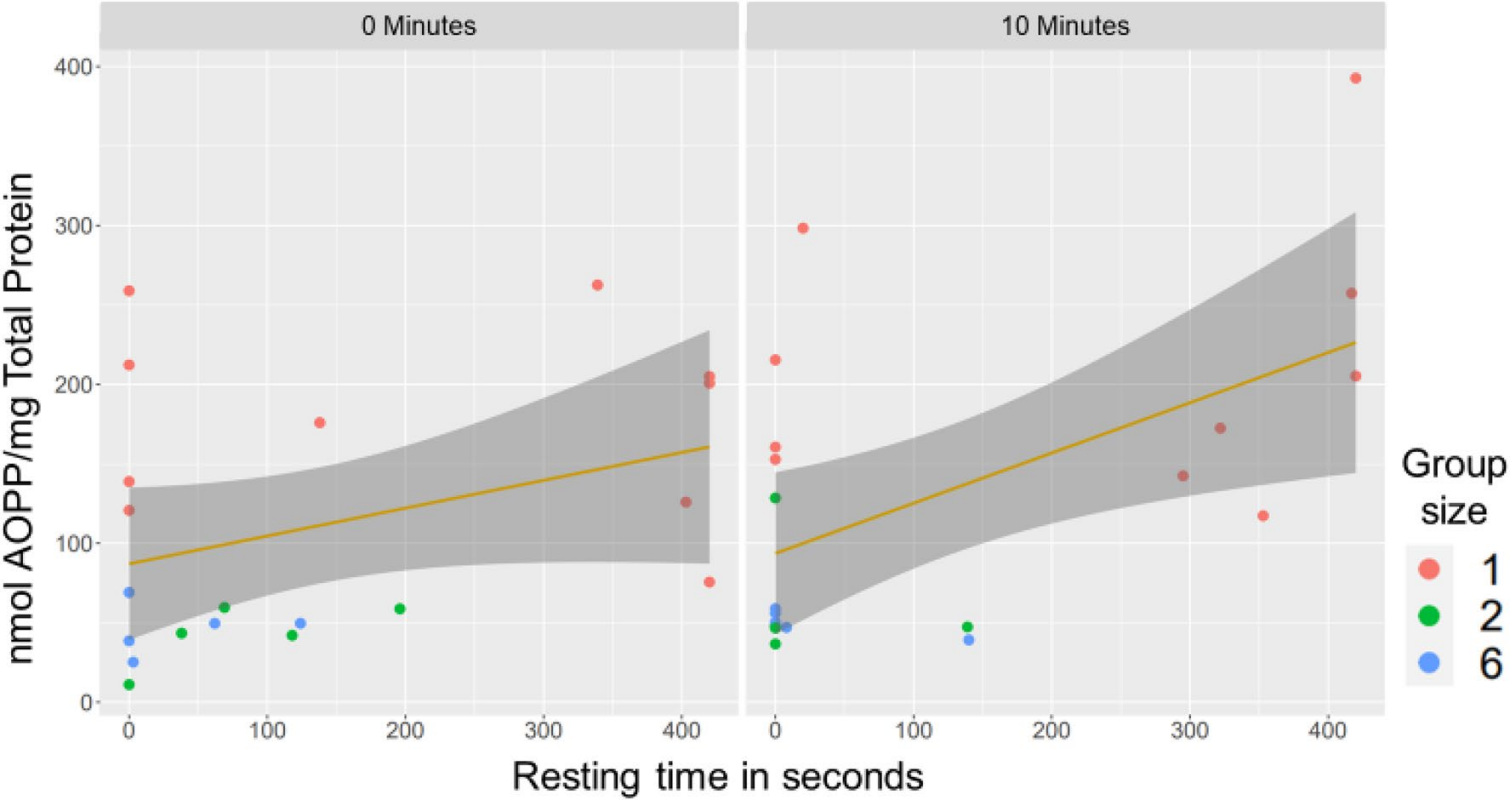
The graph displays a linear regression model to investigate the relationship between AOPP (Advanced Oxidation Protein Products) and two predictors, normalised seconds of resting time and sampling (with and without “waiting time”). The Type II tests Anova showed a significant effect on the normalised seconds on AOPP levels (F = 9.8954, *p* < 0.01), suggesting that changes in resting times are associated with changes in AOPP. However, there was no significant effect on the time point of sampling.

The linear regression analysis did not reveal a significant association between normalised resting times and cortisol or malondialdehyde (MDA) levels in fish. While resting behaviour appears to be linked to oxidative stress, as indicated by the positive correlation with Advanced Oxidation Protein Products (AOPP) levels (Fig. 6), it does not directly influence cortisol and MDA levels.

## Discussion

Our study provides new insights into the relationship between collective behaviour and stress in wild fish exposed to running water, shedding light on the unique mechanisms involved in maintaining social behaviour and reducing the stress response of group living. In contrast to the grouped fish conditions, a single individual in the flume led to a significant upregulation of stress levels, as demonstrated by physiological and behavioural responses. Therefore, the absence of conspecifics can lead to the activation of the hypothalamic-pituitary-interrenal (HPI) axis and the formation of ROS, resulting in increased cortisol levels and oxidative damage in the cells.

Previous studies have suggested that the presence of another individual can reduce stress responses in fish when dealing with environmental stressors, hypothesising that this is due to the increased calmness provided by social interactions^23, 48^. This phenomenon is commonly referred to as social buffering, which can provide individuals with the ability to cope more effectively with stressful conditions^20, 23^. By reducing stress levels, social buffering may help fish maintain normal behaviour and improve their overall well-being^38^. Conspecifics have been shown to buffer the stress response and decrease cortisol levels in several fish species^24, 49, 50^, highlighting the importance of social context in stress regulation. The availability of conspecifics may provide a sense of safety and security, leading to lower stress hormone release.

Furthermore, it has been shown that social isolation and lack of social support can negatively affect animals’ coping abilities and stress levels^50, 51^. For example, studies on social buffering in various species have indicated that being in the presence of conspecifics can reduce the physiological and behavioural responses to stress by releasing oxytocin^52, 53^. In the context of fish, research has suggested that conspecific social support can improve their ability to cope with stressors such as low oxygen levels and high water temperatures^17, 51^. While social buffering has traditionally been thought to reduce stress responses in fish, our data suggest that it may also activate other physiological responses to reduce stress. In fact, in the presence of conspecifics, social buffering may reduce stress by activating the gene expression of antioxidant enzymes, which can help mitigate the adverse effects of ROS production and reduce cellular damage. Therefore, it is essential to consider other physiological responses, such as antioxidant responses, that are directly involved in stress reduction. Integrating all these parameters can provide a much more accurate picture of the stress condition, detailing what is happening at the molecular, cellular and systemic levels.

When animals are exposed to environmental changes, they may experience an excess production of ROS, which can lead to oxidative stress and damage to biomolecules, cells and tissues^54^. The HPI axis may be activated in response to this stress, releasing cortisol as a stress hormone. Cortisol, in turn, can also stimulate the production of ROS and exacerbate oxidative stress^37^. Although it may appear counterintuitive, these exhibit characteristics of a classic positive feedback control mechanism capable of rapidly activating the antioxidant defence system. Activation of the antioxidant defence system can limit the presence of ROS while limiting oxidative stress damage.

Under the experimental conditions of our study, solitary fish demonstrated a limited ability to cope with the stress of swimming in running water, resulting in increased oxidative stress. Notably, activating genes encoding antioxidant enzymes in the solitary fish group occurred only as a recovery response after removing the stressor. This transcriptional activation was observed 10 min after the end of the treatment. The induced proteins encompassed both cytoplasmic (Prdx4) and mitochondrial (SOD2, Prdx5, GPx4) enzymes. The involvement of catalase (CAT) is particularly intriguing, with increased mRNA expression possibly linked to peroxisomal proliferation, a phenomenon commonly observed in animals exposed to stressogenic conditions^55^.

In contrast, the activation of the antioxidant system was more pronounced in the two-fish and six-fish groups, with the two-fish group displaying a more robust response. The two groups exhibited quantitative and qualitative differences in their stress responses. In the two-fish group, there was widespread gene activation of enzymes involved in counteracting excessive hydrogen peroxide production, such as Prdx2, Prdx5, and GPx4. Conversely, the six-fish group showed a more significant response against the increased formation of the superoxide anion, as evidenced by the enhanced activation of the *sod1* and *sod2* genes. Notably, the induction of superoxide dismutases (SODs) was accompanied by the induction of Prdx2 and GPx4, which fulfil complementary roles in eliminating hydrogen peroxide produced through superoxide anion dismutation in the cytoplasm and mitochondria.

A common observation in both groups was that the primary site of ROS formation appeared to be the mitochondrion, as indicated by the involvement of genes specifically expressed in this organelle (*sod2*, *prdx5* and *gpx4*)^25, 38, 40^. Of particular significance is the upregulation of glutathione peroxidase 4 (GPx4), suggesting its vital role as a supreme ROS scavenger and an essential component of the fish’s antioxidant defence system under stressful conditions^38, 41, 42^. GPx4 plays a crucial role in cellular defence against oxidative stress by detoxifying lipid peroxides generated during periods of oxidative stress. The early induction of GPx4 likely serves to cope with increased lipid peroxidation in the fish subjected to the treatment, thereby maintaining cellular homeostasis and preventing damage to cell membranes and other critical cellular components^25, 41^. GPx4 operates as a vigilant scavenger at the mitochondrial level, where an initial surge in ROS production often arises due to oxygen utilisation by cytochrome c oxidase, a vital component of the electron transfer chain. This chain is integral to ATP production via ATP synthase, and the heightened response of GPx4 underscores its significance as the frontline defender against oxidative stress in fish^56–58^.

The observed pattern in our study indicated a return to baseline gene activation levels following the treatment, suggesting effective regulation of reactive oxygen species (ROS) production. This finding implies that a dedicated recovery phase was unnecessary as the stress response was efficiently controlled. The complexity of the stress response to being in the flume was illuminated, revealing the involvement of diverse signalling pathways and cellular processes. Furthermore, the context-specific activation of specific genes in distinct cell types or tissues, influenced by various factors, highlights the multifaceted nature of the mechanisms at play. These objective findings expand our understanding of fish’s intricate stress response systems, presenting new opportunities for further investigation and exploration.

Furthermore, our study highlighted the importance of analysing stress indicators directly in the muscle tissue. Due to the direct involvement in movement and ability to monitor responses to stress and oxidative stress, myocytes are among the cells that consume the most oxygen and are, consequently, more likely to produce ROS. Moreover, stress and muscle activity can influence each other bidirectionally, whereby stress can affect movement patterns and reduce swimming ability. The use of muscle tissue as a stress bioindicator has been widely studied in larger fish species, but our study provides new insights into its usefulness in smaller fish. Measuring stress levels in muscle tissue is a good alternative in small fish, where obtaining other tissues in the amount required to carry out individual assessments is impossible. Above all, cortisol levels were positively correlated with plasma levels^31, 54^, and therefore, skeletal muscles supply an alternative matrix for analysing stress levels in smaller-sized fish with low amounts of blood or other available tissue.

Nevertheless, other studies have explored the role of the central nervous system (CNS) in fish stress responses, revealing the involvement of other tissues in the stress response^59^. Indeed, the different results between the various experimental groups suggest that early activation of the antioxidant system may not depend exclusively on the levels of ROS production. Furthermore, evidence suggests that in vertebrates, the relationships between oxidative stress, antioxidant defences and CNS function are equally central to responses to the physical and social environment^56^. Unfortunately, links between cognitive performance and oxidative status remain virtually unexplored by behavioural ecologists. In particular, it is not known whether the levels of oxidative stress found in wild animals may be related to behaviour and cognitive performance, processing information in an unpredictable or adverse environment. Although we did not explore individual differences in behaviour and stress response due to the limitations of tracking individual fish in our experiment, our findings suggest that behavioural observations, such as resting times, could provide valuable insights into the stress response of fish and should be considered as part of a multifaceted approach to studying stress in fish.

The lower resting times observed in the grouped fish than in solitary fish suggest that being in a group may provide a stimulating environment that encourages swimming and reduces the likelihood of stress-related behaviour such as restlessness or immobility. Especially as it highlights the potential importance of social factors in modulating stress responses in fish, resting times can reflect the activity level or the fish’s willingness to swim, as fish swim typically continuously in the wild, except when they need to rest or hide^60^. For instance, Dreosti et al.^61^ found that exposure to a novel environment increased the exploratory behaviour of zebrafish while decreasing their resting behaviour compared to fish in a familiar environment. Previous research shows that fish have trouble adjusting to their surroundings^5^; therefore, we cannot exclude that this might have also impacted our results. Fish depend on sensory cues to navigate and comprehend their environment, which can cause confusion and disorientation when disrupted or unfamiliar^12^. Fish may stop swimming and freeze at the downstream grid because they feel exposed, unlike the group, which exhibits bold behaviour and swims more. It is well known that the availability of companions increases swimming performance^62^. Moreover, previous studies have shown that even visual contact with conspecifics can enhance swimming abilities in fish^63^, which further supports the importance of social interactions for fish performance and well-being.

Therefore, the reduced coping capacity of solitary fish contributes to their extended resting periods in the downstream grid, as they may struggle to acclimate to the current and maintain their position. Our observation supports that the stress levels of solitary fish did not decrease after the 10-min waiting period, implying that they may have been more stressed and less able to cope with the environment than fish in groups. Although we provided the fish with a 5-min acclimation interval before recording, it is possible that the new circumstances in an open field with no shelters and flowing water exposed to predators first scared them. This phenomenon is evident in the six-fish condition involving higher capturing times over time. Hence, based on the available literature, fish can exhibit avoidance behaviour and altered swimming patterns in response to novel or stressful environments^12, 62^, which supports the possibility that the new circumstances in our study, such as an open field with no shelters and flowing water exposed to predators, may have initially scared the fish and contributed to their reduced coping capacity. While our study primarily focused on non-migrating juvenile *T. muticellus*, it is essential to acknowledge that the ecological context of this species is characterized by preferences for specific habitats, such as riffle and pool areas, as well as their selection of moderate water velocities^44^.

While many aspects of the complex relationship between stress and behaviour in fish remain to be fully elucidated, our findings hold significance for understanding how these animals respond to varying environmental conditions, which is particularly pertinent to species inhabiting dynamic aquatic ecosystems. These environments can subject fish to a range of stressors^43^, impacting their stress levels and coping mechanisms. Such insights are invaluable for devising management strategies that promote the health and well-being of fish populations especially during their juvenile stages when they exhibit specific ecological behaviours. Further research could investigate the specific mechanisms underlying the effects of social buffering on oxidative stress and antioxidant defences, particularly in the context of the CNS and how it influences physiological responses in fish. Previous research has shown that isotocin, a neuropeptide involved in social behaviour, can reduce the cortisol response to stress in fish, suggesting that social buffering may involve the CNS and modulate the HPI axis response^51, 61^. Thus, our work lays the foundation for exploring these intricate relationships and their ecological ramifications, such as antipredator behaviour, spatial distribution, refuge-seeking behaviour, and foraging dynamics, all characteristic features of juvenile cyprinid fish and hold substantial ecological relevance.

## Conclusion

In conclusion, our study demonstrates the effectiveness of utilising a physiological marker to assess the effects of collective behaviour in fish residing in running water environments. We gain valuable insights into the multifaceted dynamics between social interactions and physiological responses by evaluating the oxidative stress response in conjunction with cortisol release. This approach is a powerful tool for comprehending the mechanisms underlying group living and promoting organismal health and fitness. Although social fish rarely experience isolation in natural or aquaculture settings, our findings shed light on the potential physiological benefits of social interactions in fish. These discoveries significantly affect conservation efforts to preserve fish populations and their natural habitats.

## Material and methods

### Ethical approval

The study was conducted following the Declaration of Helsinki and approved by the Department of Economic Development Protection of Flora and Fauna of the Metropolitan City of Turin, No. 4457 of 29 October 2020, under Italian Decree-Law No. 73.of 19 March 1948, Italian Law No. 56 of 7 April 2014, Italian Law No. 114 of 11 August 2014, Regional Law No. 23 of 29 October 2015, and Italian Legislative Decree No. 26 of 18 August 2000, and by the Director of the Provincial Office for Hunting Fishing Parks and Forests of the Province of Cuneo, No. 3014 of 26 October 2020, under Regional Law No. 37 of 29 December 2006, Provincial Council Decree No. 109 of 13 March 2007.

### Animals

Juvenile Italian riffle dace (*Telestes muticellus*), an endemic fish species of Northern Italy, France, and Switzerland^44^, was used to investigate the effect of water flow velocity on fish behaviour and physiology. 100 fish with a size of 5 ± 0.5 cm were caught using electrofishing in Noce stream near Pinerolo, northern Italy, in May 2021 (44°56′18.52″N 07°23′11.24″E) and transported to a hatchery in Porte, Italy. Fish were kept in the food-rich river-fed tanks outdoors at 15.5 °C for at least two weeks before starting experiments. After this time, they were transferred indoors to acclimate to hatchery conditions for more than 30 h in habituation tanks at 14.5 °C. Fish were transferred to indoor tanks every three days, and experiments were carried out over 9 consecutive days. Individuals from the same habituation tank were employed for both two-fish and six-fish groups to minimize the potential for disruption and stress responses in fish used for subsequent experiments. Furthermore, habituation tanks were utilised no more than twice daily, ensuring that the fish remained in familiar social environments and minimising any potential disturbances to other fish awaiting experimentation.

### Experimental setup

Experiments were carried out in a portable flume with transparent Perspex walls (30 × 60 × 30 cm). A stainless-steel wire mesh grid delimited the swimming arena with an opening 1 cm downstream and a flow straightener upstream (which also served to generate a laminar flow). Mean flow velocity was controlled by a pump inverter and by varying the height of a downstream weir. Volumetric flow, water level, and temperature were monitored with dedicated sensors (AquaTransTM AT600, Baker Hughes; BUS0025, BALLUFF; PT100 thermoresistance) connected to a data logger (DAQ USB-6002, National Instruments). Two video cameras (Sony FDR-AX43; 1920 × 1080 pixels, 50 fps) were positioned laterally and beneath the flume to track fish positions.

### Experimental protocol

The experimental setup consisted of treating the fish with three consecutive water velocities: a habituation time of 5 min at a mean flow velocity of 10 cm/s, followed by 30 min of testing at increasing velocities (10, 20, and 35 cm/s, each for 10 min). Transitions between the three different flow regimes lasted 30 s. To comprehensively assess fish behaviour and stress responses under ecologically relevant conditions, we employed three consecutive water velocities, including a gradual increase, to mimic the dynamic hydrodynamic environments often encountered by fish in natural riverine ecosystems. The water level and temperature were kept constant at 15 cm and 14.5 ± 0.5 °C, respectively. Three different group sizes of 1, 2, and 6 fish were tested in a randomised order with 10 trials of the two- and six-fish groups and 20 trials with a solitary fish (Figure S1). In this study, we utilised the six-fish treatment as a reference point for physiological measurements, considering it a control situation due to its alignment with the natural group behaviour of T. muticellus observed in the wild. While physiological measurements were not conducted throughout the entire experiment, this approach allowed us to assess how alterations in group size influenced stress responses within a context that simulates the species’ typical social structure and habitat conditions. From the two and six-fish treatments, two fish were sampled from each trial for testing to achieve an equal sample size between treatments. Half of the tests were immediately transferred to an anaesthetic bath.

In contrast, the other half was kept in the flume for an additional period of 10 min without running water, which we refer to as the “Waiting” period. During this time, the fish were not subjected to any flow in the flume. Fish were killed with an anaesthetic overdose with 0.2 mL/L 2-phenoxyethanol, half directly after the treatment and the other half, including the waiting period, and skeletal muscle samples were taken from the caudal peduncle post-mortem. Samples for cortisol, advanced oxidation proteins (AOPP), and malondialdehyde (MDA) were stored at − 20 °C till analysis. Samples for qPCR analysis were placed in 5–10 the tissue volume in RNA later and kept for 12 h at 4 °C before they were transferred to − 20 °C.

### Cortisol assay

The cortisol analysis was performed following the protocol published by Bertotto et al.^33^ with a specific microtiter radioimmunoassay (RIA). For the analysis, 50 mg of tissue was homogenised in liquid nitrogen. Steroid hormones were extracted using 8 mL of diethyl ether. The radiation was measured using a beta-counter (Top-Count NXT, Perkin Elmer Life and Analytical Science, USA).

### Oxidative stress damages

Advanced oxidation protein products (AOPP) were measured using a microplate reader at 340 nm (SpectraCount AS10000, Packard, Netherlands) with a chloramine-T standard in 25 L of muscle homogenates as duplicate followed Witko-Sarsat et al.^64^. Thiobarbituric acid-reactive compounds (TBARS) were analysed following Yoshida et al.^65^ in 100 mg muscle tissue homogenised with 0.125 M Tris HCL. Malondialdehyde (MDA) levels were measured spectrophotometrically at 535 nm. Measurements were standardised with protein levels using the Thermo ScientificTM Pierce BCA Protein Assay Kit (Waltham, MA, USA).

### Primer design, RNA extraction, and cDNA Synthesis to measure antioxidant enzyme expression

Sequences from the NCBI database were used to create the primers, which MUSCLE then aligned. According to a BLAST comparison, the most closely related species was determined to be Pimephales promelas. The IDT Oligo Analyzer tool was used to analyse the primer sequences, and primers were designed in the coding areas.

The RImeZOL TM reagent (Canvax, Córdoba, Spain) was used for RNA extraction following the manufacturer’s instructions. Using a BiotechrabbitTM cDNA Synthesis Kit (Berlin, Germany) operating at 50 °C for 1 h + 99 °C for 5 min, cDNA was synthesised. 50 ng of cDNA and GRS Taq DNA polymerase were used in the PCR experiments (Grisp, Porto, Portugal). The PCR program was 95 °C for 5 min and 40° (95 °C for 30 s, Ta for 30 s, 72 °C for 30 s), with the final elongation at 72 °C for 5 min.

### qRT-PCR analysis

Five muscle samples from each group were subjected to a real-time qRT-PCR assay without waiting time. The housekeeping gene Gapdh was used as a control and amplified with species-specific primers to account for variations in cDNA synthesis and PCR amplification processes (Table S1). The qPCRBIO SyGreen Mix Separate-ROX kit (PCR Biosystems, Wayne, PA, USA) was used to do qRT-PCR amplifications using the following protocol: 95 °C for 2 min, 40 (95 °C for 20 s, and 60 °C for 60 s), and then the dissociation stage 95 °C for 15 s, 60 °C for 1 min, 95 °C for 15 s, and 60 °C.

### Video analysis of fish swimming

The time spent by the fish resting at the downstream grid was quantified using video recordings. Resting fish were identified as those in the region close to the downstream grid who were not moving their tail for at least one second, either laying on the grid or exploiting corners with the head facing the stream flow. The first three minutes after each water velocity change were discarded from the analysis to remove any potentially disturbing effect on the fish behaviour. The total time of fish resting was normalised by the number of fish in the flume.

### Statistical analysis

The statistical analysis was performed using R software version 4.1.0 with a significance threshold set at *p* = 0.05. To assess normality, Shapiro–Wilk, and log-transformed methods were applied as necessary. A one-way analysis of variance (ANOVA) was conducted to analyse cortisol, MDA, and AOPP levels, with the Student–Newman–Keuls test used to determine the significance among means. Antioxidant enzyme levels were analysed with a Kruskal–Wallis one-way analysis, followed by Dunn’s test for posthoc analysis if significance was found, to evaluate potential differences between their means.

Data on resting behaviour was not normally distributed and was analysed with a generalised linear mixed effects model (glmer) with a binomial error distribution fitted with a logit function using the lmer4 package in R. The number of seconds (normalised by the number of fish in the group) spent resting or swimming by the fish was used as the binomial variable. Water velocity, group size and their interaction were used in the model as predictors. A variable accounting for the experimental groups of fish examined in the same experiment with the three water velocities was input as a random factor to account for data independence. An observation-level random variable was introduced to the model to adjust for overdispersion^66^.

### Supplementary Information

Supplementary Information.

## Data Availability

The data underlying the findings of this study are available upon reasonable request. Researchers interested in accessing the data can contact the corresponding authors.
