# Supplementary material for: Social buffering of oxidative stress and cortisol in an endemic cyprinid fish
Source: Sci Rep. 2023 Nov 23;13:20579. doi: 10.1038/s41598-023-47926-8 (PMC10667237; doi:10.1038/s41598-023-47926-8)
Supplement: Supplementary file 1 — Supplementary Information. [file 41598_2023_47926_MOESM1_ESM.docx]

**Supporting Information for**

Social Buffering of Oxidative Stress and Cortisol in An Endemic Cyprinid Fish

Sophia Schumann, Gloria Mozzi, Elisabetta Piva, Alessandro Devigili, Elena Negrato, Andrea Marion, Daniela Bertotto, Gianfranco Santovito

Email: gianfranco.santovito@unipd.it

**This Word file includes:**

**Figure S1** Experimental setup

**Supporting text**

**Figure S2** on the impact of electrofishing

**Figure S3** on the fish fractions resting during experiments

**Figure S4** gene expression of specific genes without the waiting period

**Table S1** indicates primers used in the experiments


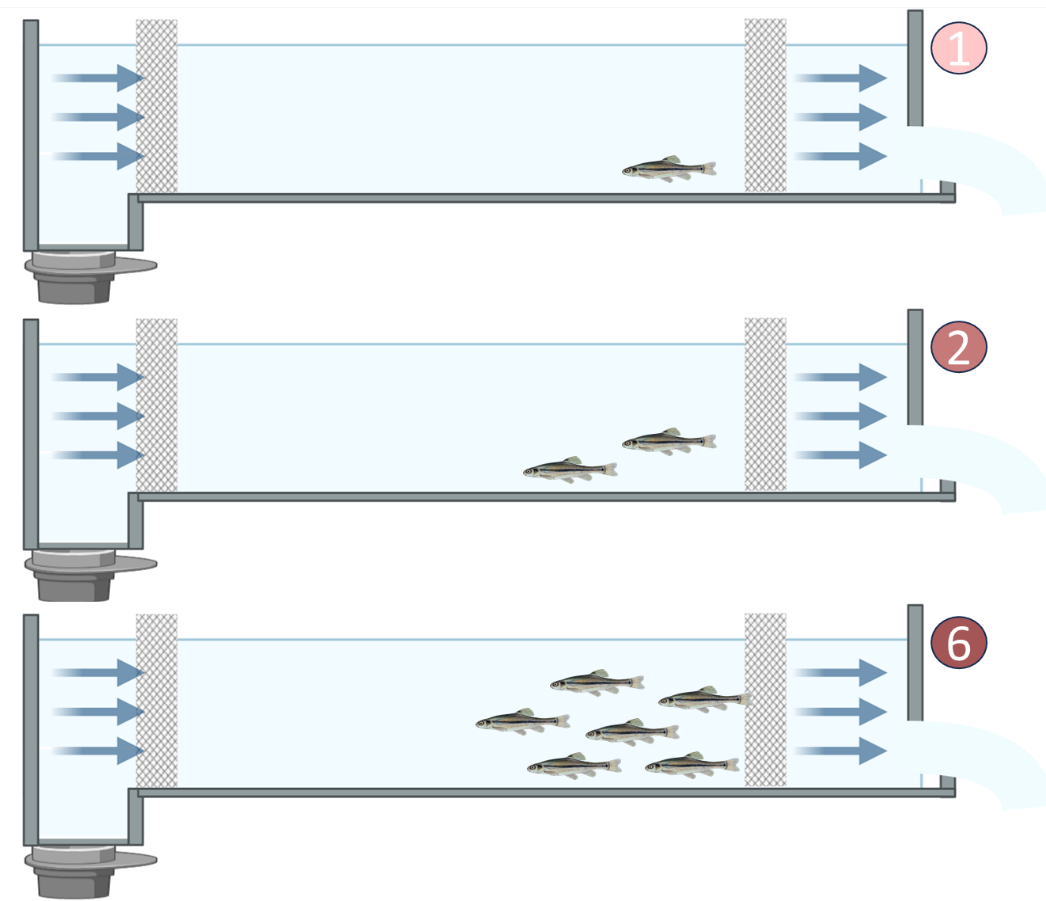


**Fig. S1.** The experimental setup consisted of three different group sizes (1,2, and 6 fish) tested in a randomised order.

Excluding the impact of electrofishing and transport on the Cortisol stress response

**Background.** In the context of scientific research, electrofishing is a valuable and less invasive method for monitoring fish health and capturing fish for experimental purposes. Unlike traditional netting or trapping techniques, electrofishing offers non-lethal sampling, ensuring fish capture and subsequent release without causing substantial harm or mortality (1). With its selective capabilities, electrofishing allows to precisely target specific species, size classes, or age groups, facilitating the collection of more precise and targeted data (2) while offering low habitat disturbance in aquatic environments compared to other sampling methods. In a preliminary setup, we aimed to investigate the potential impact of electrofishing, handling, and transport stressors on cortisol levels and explore appropriate fish handling and post-electrofishing recovery procedures. These considerations are essential for ensuring the validity and applicability of our scientific findings.

In a preliminary experimental setup on April 27, 2021, *Telestes muticellus* were captured from the same stream as the fish used in our experiments. We used bigger-sized fish to reduce the environmental effect of our study. This allowed for non-invasive blood collecting and subsequent release of the fish into their natural habitat. A single pass of backpack electrofishing was conducted using a backpack electro-fisher (Hans Grassl GmbH, Germany) in the wadable habitat (depth < 1 m), where fish were collected using a net with continuous current (approximately 560 V, 1.8 A). Following the electrofishing procedure, the fish were promptly transported to the hatchery for sampling, marking the starting point of our study (timepoint 0) during arrival at the hatchery. For accurate measurements, the fish were anaesthetised in a water bath containing 0.2 mL/L 2-phenoxyethanol before obtaining blood samples from the caudal vein.

To facilitate serum separation, the collected blood samples were refrigerated at 4 °C for 12 hours to allow clot formation. Subsequently, we performed centrifugation at 13000 rpm for 15 minutes to separate the serum from the clotted blood cells. These serum samples were then stored at minus 20 degrees Celsius until analysis. For the specific cortisol analysis described in our materials and methods section, we utilised serum volumes ranging from 20 to 50 microliters. The results are displayed in Fig. S. 1. Results suggest stabilising cortisol levels measured in the blood after 60 hours. Therefore, it is very likely that the specimens used for experimentations were not stressed anymore. Notably, cortisol sampling at 12 and 60 hours was conducted at night due to diurnal variations in cortisol levels in vairone.


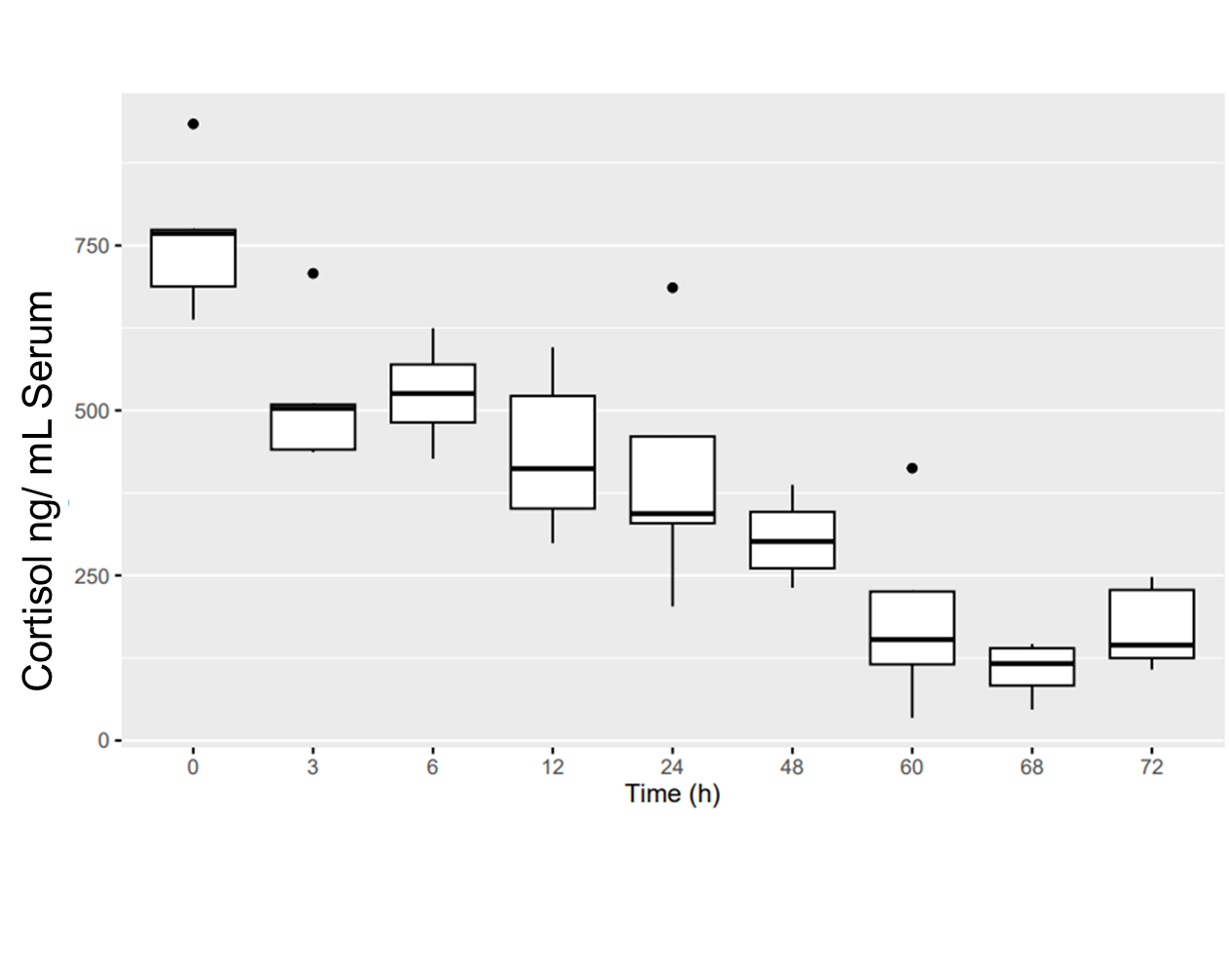


Fig. S2. Boxplot illustrating the cortisol levels measured at various time points in vairone. The boxes represent the interquartile range (IQR), with the median indicated by the horizontal line inside the box. Whiskers extend to the minimum and maximum values, while individual data points beyond the whiskers are considered outliers (N = 7 per timepoint). The results showed significant decreases in cortisol levels in vairone after 48 hours, 68 hours, and 72 hours compared to the measurement at timepoint zero (p < 0.001). Additionally, cortisol levels were significantly lower at 68 hours and 72 hours than those at 3 hours and 6 hours (p < 0.05).

Addition to Exploration of resting times during experiments

Understanding the behavioural responses of organisms is crucial for comprehending their physiological and stress-related dynamics in different experimental conditions, displacing group dynamics over different experiments (Figure 6). Our qualitative analysis of resting behaviour in fish aligns with our linear regression analysis findings. The bar graph demonstrates that fish in the single-fish treatment exhibited longer resting times and a higher percentage of fish resting than the two and six-fish treatments, indicating that social context can influence resting patterns and potentially impact stress levels.


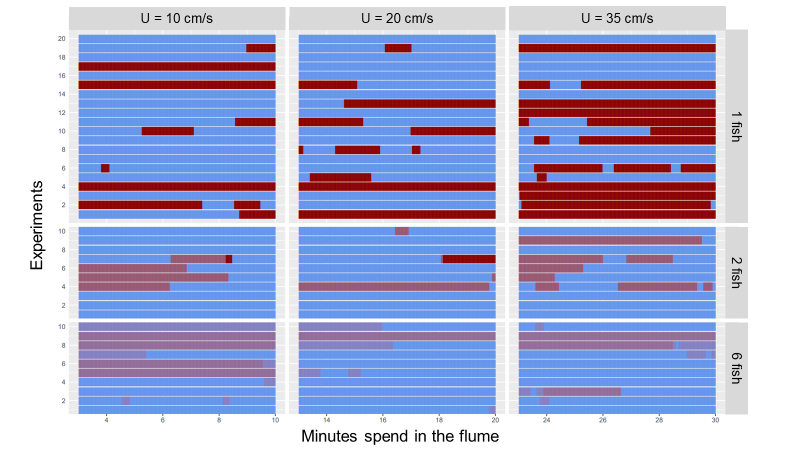


**Fig. S3.** The bar graph displays the fractions of fish resting during the experiments, labelled by number (20 repetitions for single fish; 10 repetitions per experiment for two and six-fish treatments). The intensity of the red colour represents the percentage of fish resting. The graph reveals that fish in the single-fish treatment spent more time resting than in the two and six-fish treatments, with a higher percentage of fish resting. Specifically, the single fish treatment had a mean resting time of X seconds with Y% of fish resting. In contrast, the two and six fish treatments had mean resting times of Z and W seconds, respectively, with lower percentages of fish resting. This difference was statistically significant, with p < 0.05.


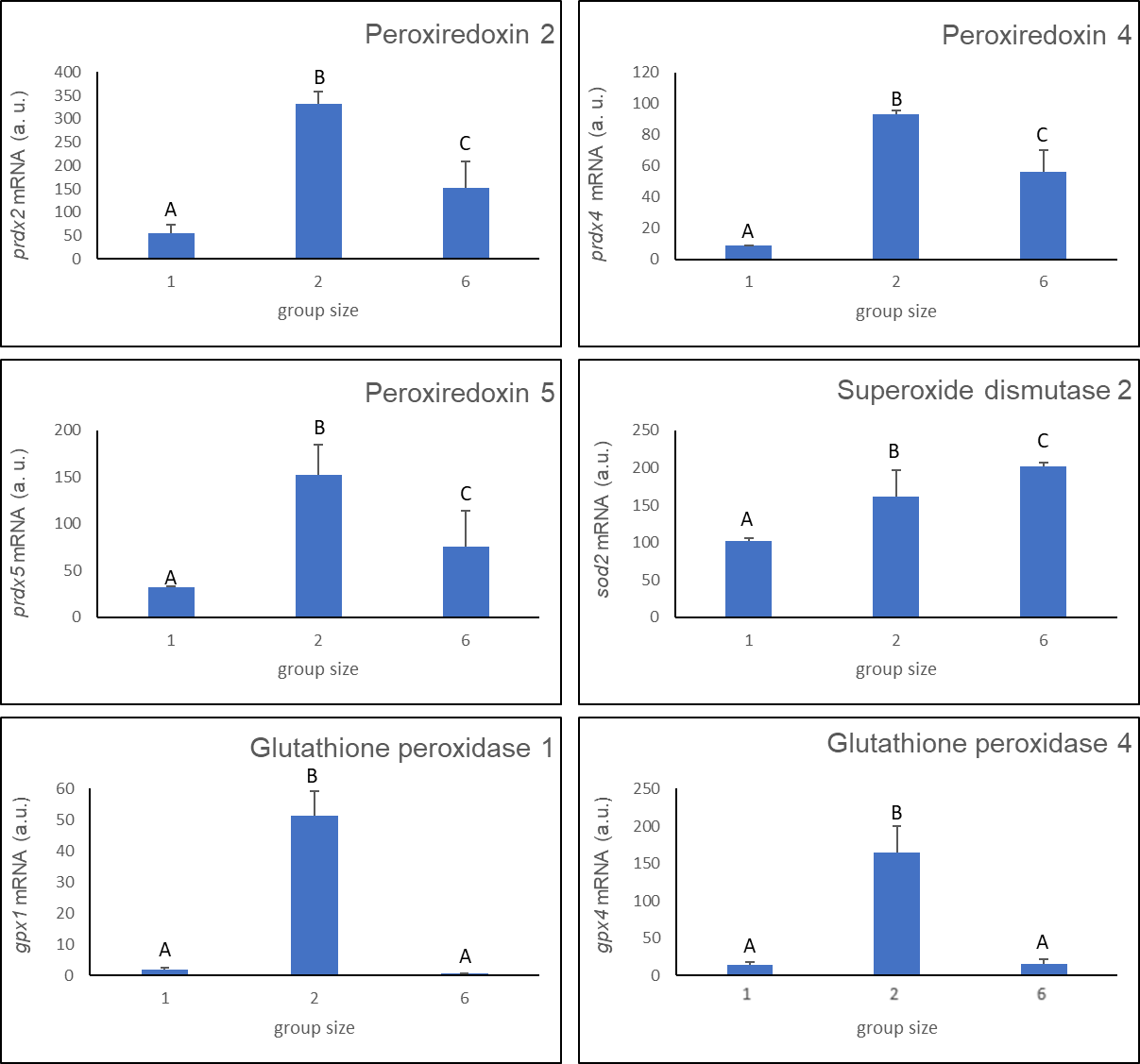


**Fig. S4.** The bar plots show the most upregulated genes directly after the treatment. Displayed are prdx2, prdx4, prdx5, sod2, gpx1 and gpx4 mRNA levels of five fish per treatment. Values (arbitrary units) are indicated as mean ± SD. Transcription levels were normalised to the gapdh housekeeping gene. Different letters correspond to significant statistical differences (p < 0.05) among different sites (Student–Newman–Keuls test).

Table S1. Primers used for RT-PCR and qPCR analysis of *T. muticellus* gene expression levels

| **Gene** | Function | **Forward primer** | **Reverse Primer** | **Product size** |
| --- | --- | --- | --- | --- |
| *CAT* | Catalase | TTTGGGATTTCTGGGGTTTGC | TGAAAGTGTGCGATCCGTATCC | 117 |
| *GPx3* | Glutathione peroxidase-3 | GGACCCAGTACATCCCATTCTCC | TGTCTCCAGGCTCTTGTTTCCCA | 186 |
| *GPx4* | Glutathione peroxidase-4 | TGCTGAGAAAGGTTTACGCA | TTGTTCCTTCAGCCACTTCC | 181 |
| *Prdx1* | Peroxiredoxin-1 | GAAGGTCCCTCTGGTGGCAGA | ACTGGCAGGTCATTGATGGTT | 147 |
| *Prdx2* | Peroxiredoxin-2 | ACTCGGCTCCATGAACATCCCT | TCGTTGATGGTGATCTGCCTCA | 150 |
| *Prdx4* | Peroxiredoxin-4 | GGCACTGCTGTTGTCAATGGA | TCTGAGTGTGTGTCCCTGATCCTC | 158 |
| *Prdx5* | Peroxiredoxin-5 | TGGGGAAAGGAGAATGGAGCAGA | GACCACTCCGTCTTCAATCAGCA | 165 |
| *Prdx6a* | Peroxiredoxin-6a | TCACTGCCCGTTGTGTGTTTGTG | TCTTGACCAGGCTTCCAGTCCAC | 172 |
| *Prdx6b* | Peroxiredoxin-6b | ACGTGCTGGCATTTCCCTGTAAC | TATTCCGACCACAGCGATCTTGC | 131 |
| *sod1* | Superoxide dismutase 1 | AGCGACTCGTCTCCAGTGAA | TTGTGAGGGTTGAAGTGCGG | 134 |
| *sod2* | Superoxide dismutase 2 | CACTACAGGTCTCGTCCCAC | CCCAGCTCACAACATTCCAG | 116 |
| *GAPDH* | Glyceraldehyde 3-phosphate dehydrogenase | ATCACAGCCACACAGAAGAC | AGGAATGACTTTGCCCACAG | 126 |

**SI References**

1. S. Awata, T. Tsuruta, T. Yada, K. Iguchi, Stress hormone responses in ayu Plecoglossus altivelis in reaction to different catching methods: comparisons between electrofishing and cast netting. *Fish Sci* **79**, 157–162 (2013).

2. S. M. Reid, T. Haxton, L. F. G. Gutowsky, Boat-electrofishing transect location and flow levels: influence on riverine fish monitoring in non-wadeable habitats. *Environ Monit Assess* **193**, 680 (2021).
